# Turnover intention and coping strategies among older nursing assistants in China: a qualitative study

**DOI:** 10.3389/fpsyg.2023.1269611

**Published:** 2023-09-29

**Authors:** Yuting Tan, Qian Zhao, Huafeng Yang, Shufen Song, Xiaohua Xie, Zhiying Yu

**Affiliations:** ^1^Department of Gynaecology, The First Affiliated Hospital of Shenzhen University/Shenzhen Second People’s Hospital, Guangdong, China; ^2^Department of Functional Neurology, The First Affiliated Hospital of Shenzhen University/Shenzhen Second People’s Hospital, Guangdong, China; ^3^Department of Nursing, The First Affiliated Hospital of Shenzhen University/Shenzhen Second People’s Hospital, Guangdong, China

**Keywords:** older nursing assistants, eldercare institution, turnover intention, coping strategies, phenomenological analysis, qualitative research

## Abstract

**Introduction:**

With the increasing aging population, older nursing assistants have made significant contributions to institutional eldercare. However, there is a high turnover rate among these workers, and it is crucial to address this issue and find ways to stabilize the workforce. This study aimed to explore the factors influencing turnover intention and coping strategies among older nursing assistants, in order to provide targeted assistance and guidance to reduce their intention to resign and ultimately lower the turnover rate.

**Methods:**

Qualitative research methods were employed to conduct semi-structured interviews with older nursing assistants in Changsha. The data obtained from these interviews were then analyzed using a phenomenological analysis approach and NVIVO (QSR International, Doncaster, Australia) software version 11.0.

**Results:**

It is found that several factors influence turnover intention among older nursing assistants. Which include work pay, work environment, professional identity, external motivation, and work pressure. Additionally, the coping strategies employed by these individuals in relation to their intention to resign include self-regulation, seeking support, self-improvement, and exploring motivation.

**Discussion:**

It is also evident from our study that reducing the turnover intention of older nursing assistants requires a collaborative effort from older adult care institutions, functional departments, and eldercare nursing assistants themselves. By addressing the factors influencing turnover intention and providing support and resources for coping strategies, we can work towards stabilizing the workforce and improving institutional eldercare.

## Introduction

China’s aging population ranks first in the world, moreover, the problem of rapid aging in China has attracted global attention (Nie et al., 2021). Aging is further burdened with poverty, disease, disability, service and care limitations (Jeong and Lee, 2020). Currently, there are various modes of old adult care in China, including self-care, family care, institutional care, and community care. With economic development, changes in family structures, and evolving attitudes towards eldercare, more and more older adults individuals are willing to choose institutional eldercare. Institutional eldercare is an important mode for global older adult care, and the quality of care services heavily relies on the talent and expertise of the older nursing assistant workforce (Hanratty et al., 2018; Xing et al., 2018). It is important to note that older nursing assistants are not synonymous with registered practical nurses. Instead, they are professionals employed by social service organizations to provide dedicated care and comprehensive nursing services to the older adults (Beswick et al., 2008). The “Regulations on the Management of Older Adults Care Institutions” issued by the Ministry of Civil Affairs in 2013, explicitly stated that older nursing assistants employed in eldercare institutions should possess professional and technical qualification certificates issued by the Labor Bureau to be eligible for employment. Therefore, this certification ensures that older nursing assistants undergo professional skills training and pass assessments before being certified for employment. Additionally, the regulations emphasize the importance of regular training on professional skills and ethical education for staff members in eldercare institutions.

The workforce of older nursing assistants has made significant contributions to China’s institutional eldercare, and the construction of this workforce is crucial for improving the quality of older adult care services. While challenges in eldercare institutions are prevalent worldwide, they typically manifest as severe shortage and high turnover rates among older nursing assistants (Lundmark et al., 2021). This imbalance between supply and demand has resulted a shortage of both quantity and quality in older nursing assistant workforce. Various factors influence older nursing assistants’ decision to leave their nursing homes or the nursing care industry (van Teunenbroek et al., 2020). Common reasons include low salary, high work intensity, high work pressure, poor professional sense of belonging, and limited social recognition (Tuckett and Oliffe, 2016; Xiong et al., 2018). Studies have demonstrated that negative emotions such as anxiety and depression are more common among older nursing assistants than other general nurses (Xu et al., 2022; Bergqvist et al., 2023). These factors mentioned above affects the physical and mental health of older nursing assistants, leading to reduced job satisfaction and lower quality of nursing services (Dwyer et al., 2017; Melo et al., 2020). Turnover intention refers to an individual’s inclination to change jobs in the future. It is an precursor to resignation and serves as a reliable predictor of actual turnover (de Oliveira et al., 2017; Cardoso et al., 2020). The shortage of older nursing assistants, high turnover rate and high mobility remains significant challenges in eldercare institution (Drennan and Ross, 2019). It is estimated that by 2035, the population aged 65 yrs. and above in China will reach 310 million and by 2050, it will reach 380 million, accounting for 22.3% and 27.9% of the total population, respectively (Li et al., 2022). With such an inexorably increase in the aging population, the demand for long-term care will also increase significantly. The high turnover rate among older nursing assistants is not only limited to China, but also is a global phenomenon, especially in developed countries such as United States and Japan (Abdi et al., 2019; World, 2021). Therefore, understanding、timely guiding and counseling older nursing assistants’ turnover intentions requires urgent attention.

Coping strategies plays a significant role in how individuals handle or manage stress, challenges, or difficult situations in their work. We found that the majority of research focused more on the institutional and societal aspects of eldercare, with less emphasis on studying the coping attitudes and behaviors of nursing assistants from an individual perspective. For instance, the present research has highlighted the importance of creating healthy psychosocial work environments and implementing disability prevention management interventions as organizational concerns for the employees (De Wind et al., 2014). Stable working teams, with adequate staffing, may also contribute to a reasonable workload and work pace (Sousa-Ribeiro et al., 2022). Previous studies in this area employed a quantitative approach and only highlighted factors that influencing resignation after older nursing assistants have already left their jobs at eldercare institutions (Rosen et al., 2011). Actually, if we can timely understand their intention to leave, as well as gain insights into the efforts, challenges, difficulties and pain faced by older nursing assistants in the coping process, we can provide more targeted assistance and guidance to reduce their intention to leave and ultimately lower the turnover rate. Qualitative research can provide valuable insights into understand the real reasons behind the intention to leave and the coping strategies from the perspective of older nursing assistants in eldercare institution. The findings from this study informed the development of a systematic management system for older nursing assistants, providing valuable insights for their improvement and support. By understanding their experiences and challenges, appropriate measures can be implemented to enhance their job satisfaction, well-being, and retention in the eldercare workforce.

## Materials and methods

Phenomenological analysis was employed in this qualitative study. Individual in-depth interviews were conducted using a semi-structured interview outline, and Colaizzi’s 7-step analysis was used to organize and analyze data. The interview outline was designed to explore the underlying reasons behind their intention to leave the profession and understand the strategies they adopt to cope with this dilemma.

### Study participants

This study was conducted in 8 eldercare institutions located in Changsha City, Hunan Province, China, from August to December in 2020. The participants were selected through a purposive sampling method ensuring maximum variation of characteristics information such as sex, age, education level and years of experience etc. Therefore, we invited nurses from eight eldercare institutions to enroll in the study. The inclusion criteria were as follows: (1) willing to participate in research; (2) holds the older nursing assistants certificate issued by the Labor Bureau; (3) have more than 1-year work experience; and (4) provide direct care for the older adults. The exclusion criteria included (1) who has not submitted a resignation letter to the ministry of manpower and has not expressed any intention to leave to the researchers; (2) who has already resigned, and not working within the eldercare organizations where the study was conducted; and (3) the research subjects were participating in other studies that may introduce bias to the results of this research.

### Develop an interview outline

Based on the research purpose, after reviewed the literature and consulted with members of the research team and experts in nursing management, a final guiding question is formulated in this study. These questions aim to explore the perceptions, feelings, job satisfaction, turnover intention, coping strategies, and future work plans of the participants. The guiding questions are as follows: ① How do you perceive your work as a nursing assistant in the eldercare institution? ② Can you talk about your feelings and emotions while working in the eldercare institution? ③ Do you like your job as a nursing assistant? If yes, what are the reasons for your liking? If not, what are the reasons for your dislike? Have you ever considered resigning? If so, what were the reasons behind your consideration? ④ Based on your experiences, how do you cope with your turnover intention as a nursing assistant? and ⑤ When you encounter difficulties at work, how do you handle them? Have you received help from others? These guiding questions will serve as a framework for the semi-structured in-depth interviews with the participants, allowing for a comprehensive exploration of their experiences and perspectives.

### Data collection method

The semi-structured in-depth interviews was conducted with older nursing assistants using guiding question ① ~ ⑥. To ensure a comfortable and open environment, the interviews were scheduled at a convenient time for the participants, free from interruptions. The interviews took place in the caregivers’ lounge, which were equipped with comfortable seating and refreshments. As researchers who were familiar with the participants’ work environment, we established a good rapport by expressing our concern and greetings. Prior to the interview, the research purpose and duration was explained to the participants, and they were informed that the entire interview process would be recorded. They were also encouraged to promptly report any discomfort during the interview. Respect for the older nursing assistants is of utmost importance, as they are vital to gaining their understanding and support. The research process and results were strictly adhering to confidentiality principles to protect the participants’ personal identity and sensitive information. Measures were implemented to ensure the anonymity of the data, and only the research team had access to and handle the personal information of the participants. It was crucial for researchers to refrain from disclosing the specific turnover intentions and related statements of individual nursing assistants to the leadership of the eldercare institution. This was to prevent any potential pressure or unfair treatment that the participants may face in their future work. The collected information was being used solely for research purposes and was be destroyed promptly after the study concludes. Informed consent was obtained from the participants before the formal interviews commence. During the interviews, attention should be paid to observing non-verbal behaviors such as facial expressions and gestures, which was be recorded. The participants were not allowed to ask any leading questions. The sample size was determined based on information saturation. This study has obtained approval from the Ethics Committee of the Second People’s Hospital of Shenzhen (Approval No: 2020081202).

This study employed several measures to ensure the reliability and validity of the data analysis. Firstly, all equipment such as interview outline, pens, and recording devices were checked to ensure they were functioning properly before the study. During face-to-face communication with the interviewees, notes were taken, and observations were made simultaneously. After the interviews, the recorded audio was reviewed to ensure clarity and authentic. The interview materials were organized and stored in a categorized manner as soon as finishing each interview.

### Data analysis

This study used Cross-check data to enhance the reliability and validity for data analysis. The interviewer transcribed the interview content into written form within 24 h after the interview, which was be reviewed by another researcher to ensure accuracy and completeness. Two researchers were independently analyzing the data using the Colaizzi’s 7-step analysis method. The Nvivo11 software (QSR International, Doncaster, Australia) was be used for coding and categorization. In case of any discrepancies, any discrepancies that arose during the analysis was be resolved through consultation or discussion within the research team. The Colaizzi’s 7-steps were as follows: ① all the information was read carefully, ② important statements were extracted, ③ recurring opinions were coded, ④ the coded opinions were collated and aggregated into themes, ⑤ detailed descriptions were written without eliminating any information, ⑥ similar opinions were identified, and ⑦ the responses were returned to the participants for verification. For this purpose, the interviews were converted into text within 24 h of every interview. The semantic unit (described as the turnover intention and coping strategies in words, sentences, or paragraphs) was listed. After the text from the interviews was encoded, the nodes (the unit described using general concepts and phrases) were reviewed to identify themes according to the similarities and differences in the code integration as well as the similarities and correlations for the extension category. Finally, the categories were checked and the data were used again to ensure the strength of the theme identification code.

## Results

### Participants’ baseline characteristics

The final study sample included seven certified older nursing assistants aged from 21 to 55 years, who worked in different attributes eldercare institutions (publicly or privately). This sample size was considered appropriate for the identification of commonalities and divergences among participants. It is worth noting that just one of the interviewees was men, which reflects the gender distribution in the occupation, which is predominantly female in China. Older nursing assistants’ baseline characteristics was shown in Table 1.

**Table 1:** Baseline characteristics information of participants.

Number	Sex	Age	Education level	Years of experiences	Marital status	Attributes of work unit	Interview time
A	Female	21	Technical secondary school	1	No	Public	60 min
B	Female	47	High school	5	Yes	Public	55 min
C	Male	52	primary school	3	Yes	Private	48 min
D	Female	38	primary school	8	Yes	Public	65 min
E	Female	55	Junior high school	12	Yes	Public	50 min
F	Female	31	Junior high school	6	Yes	Private	60 min
G	Female	49	Junior high school	4	Yes	Private	65 min

**Figure 1:**
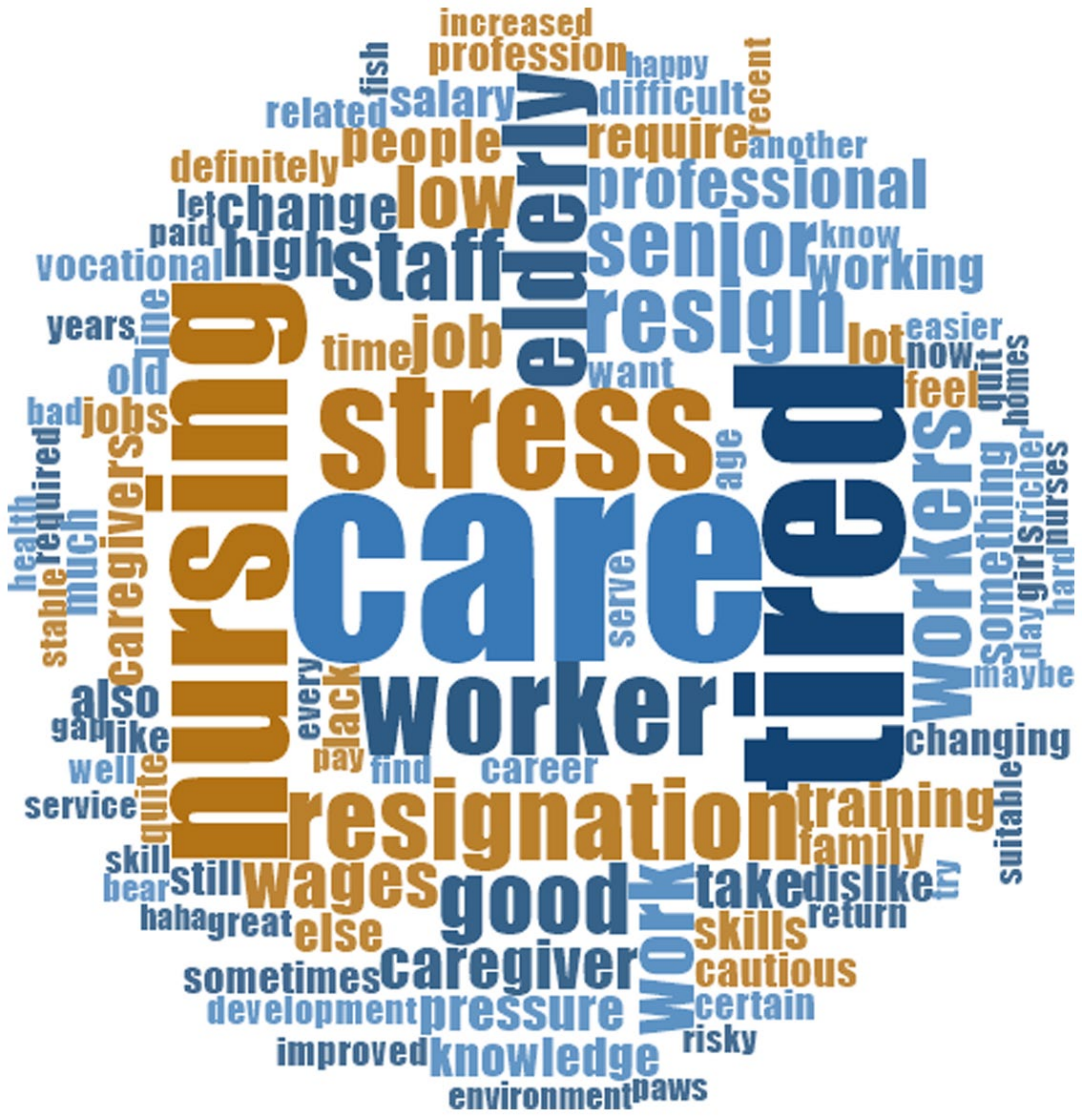
Word cloud of word frequency of interview text.

Firstly, to visually represent the word frequency in the recorded text, a word cloud was created using the word frequency function in NVIVO software version 11.0. The size of each word in the word cloud corresponds to its frequency of occurrence. As shown in Figure 1, the larger the area of the text in the figure, the higher its frequency of occurrence. After integrating and screening the node, 11 tree nodes and 13 sub-nodes were constructed. The information pertaining to the node levels and reference points are listed in Table 2. Through an analysis of the number of sources of node materials and the number of reference points, the primary and secondary roles of each node in stimulating the turnover intention among older nursing assistants were judged. According to the number of reference points, we determined that the top five nodes in descending order were pay, job responsibilities, unexpected risk, social recognition, and employment opportunities (Table 2).

**Table 2:** Themes and supporting quotes.

**Themes and sub-themes**	Number of node material sources^a^	Reference points^b^	**Supporting quotes**
1. Vacation	4	11	
Vacation time	2	6	Very few holidays, lack of people to work, 2 days off a month
Vacation arrangements	3	5	It’s hard to ask for leave if you do not focus on holidays, take turns
2. Work pay	7	36	
Salary	7	15	The salary is low, except for expenses, not much money can be saved
Social Welfare	7	14	The private sector is worse than the public sector, there is no guarantee and five social insurances
Wages and benefits	5	7	Sometimes the company will be in arrears of wages, and it will be issued every long time.
3. Humanistic environment	6	20	
Interpersonal relationship	6	12	There are too many people to dealing with, they are indifferent
Department atmosphere	6	8	Bored, lifeless, nervous, stressed
4. Physical environment	4	12	
Hygiene	2	9	Dirty, strong smell, not clean
Location	2	3	It is far from the city, not very convenient
5 Social recognition	7	30	Most people have professional prejudice towards this job and do not respect us
6. Value realization	5	14	Mechanical, repetitive work, no future
7. Job responsibilities	7	35	The workload is heavy, things are too complicated, all aspects
8. Unexpected risk	7	33	
Risk of pressure ulcers	7	7	Older and sick, long-term lying in bed for fear of bedsores
Risk of falling	4	9	If you do not pay attention, you will be in trouble if her or his fall on the ground
Risk of loss	6	8	He has hands and feet, if he lost, it is my responsibility
Improper operation	7	9	Some operations are still very dangerous
9. Employment opportunities	5	27	Nowadays, worker like Yuesao and Prolactin division expert are very popular
10. Training route	4	15	Training institutions are already relatively standardized to have Yuesao or Prolactin division expert certificates
11. Competitive advantage	4	16	I am healthy and hard-working, i do not worry about to find another job

Remarks: ^a^ refers to the number of interview materials containing the node; ^b^ refers to the source of the node in all interview materials. The blue shades are the “themes”, and the white shades represents the “sub_themes” part.

**Figure 2:**
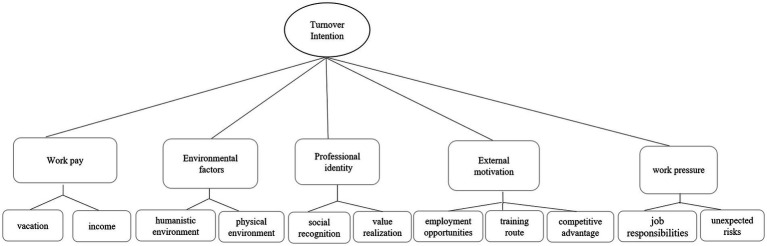
The relationship diagram of the five themes of influencing factors of turnover intention for older nursing assistants.

The integrated nodes were summarized using Colaizzi’s 7-step analysis method and five themes affecting turnover intention were identified. The derived relationship diagram is shown in Figure 2.

Each theme is described below, supported with original quotations.

### Theme 1: turnover intention

#### Work pay

As workers, older nursing assistants are entitled to take vacations and obtain labor remuneration and welfare guarantees in accordance with the law. However, their financial situation is usually insufficient to cover their living costs, and the low salary is a central factor influencing their decision to have turnover intention. While the job benefits of this profession are not optimistic, resulting in low job satisfaction.

“As you know, prices are rising and the value of the CNY (RMB) is also depreciating, but our salary have not increased much. In the last two years, our salary has only been 2,200 RMB (sigh, frown).” **(Participant A)**

“Our public welfare benefits are worse than before. There is no insurance and our income is lower than that of private ones.” **(Participant D)**

#### Environmental factors

The environmental factors included both human and physical environments. On one hand, most older nursing assistants feel that the work environment is rather poor and messy. On the other hand, older adults care nurses actually have to deal with various relationships, which can be challenging and difficult for them. Participants also reflected upon possible organizational strategies that may promote a cleaner and healthier work environment and facilitate a more pleasant and relaxed atmosphere.

“It is not easy to stay here. Some older adults have strokes or have trouble with their legs and feet, so they urinate and defecate in bed, which creates a foul smell.” **(Participant C)**

“I wanted to quit my job recently. It is not just about being tired from taking care of six or seven older adults in a row, but the problem is that the relationship is very complicated.” **(Participant F)**

“We are supervised by nurses or family members anytime and anywhere, there is no freedom.” **(Participant B)**

#### Professional identity

The participants expressed a general feeling of low social recognition and lack of self-realization in their profession, which made it difficult for them to feel a sense of accomplishment. They also mentioned that they often faced negative perceptions from their own families regarding their job.

“I will not tell my family what my job is because they will also think this job is dirty and tiring and will try to convince me to change my job.” **(Participant B)**

“Most people who do this job are women. I came here to make a living.” **(Participant C)**

“I think that there is little technical content in this job. We just need to acquire a small amount of knowledge and skills, and then we can start. It does not have much long-term significance.” **(Participant D)**

#### Work pressure

The work was described as physically demanding, but the focus was more on the mental demands. Participants mentioned constant time pressures, which led to frustration and affected the quality of care. This, in turn, made them feel inadequate and increased their stress levels. Heavy job-related responsibilities and high chances of unexpected risks also added to the work pressure for the nursing staff.

“In addition to taking care of all aspects of the older adults patient’s lives, we need to cooperate with doctors and nurses to perform simple clinical operations, such as medicine, nasal feeding, and assistive treatment.” **(Participant C)**

The participants also highlighted the potential health risks associated with their work due to the high job demands and stress levels.

“Things are very complicated. For example, basic life care must be performed carefully. Older adults are usually physically weak. If their bedding and clothing are often damp and messy, and if they do not turn over regularly, pressure ulcers are likely to occur.” **(Participant A)**

“Some of them can walk on their own. If they accidentally go out and get lost, it means big trouble!” **(Participant G)**

#### External motivation

The participants in this study expressed a declining interest in being an older nursing assistant due to the drawbacks of the profession. They cited job opportunities with higher incomes, greater flexibility, and easier workloads as reasons for this decline. However, some participants were still interested in pursuing further training to become a Yuesao or a prolactin division expert.

“My colleague has applied to a training course to become a *Yuesao* or a prolactin division expert. This training course is reliable, and I am ready to sign up.” **(Participant D)**

“I am getting older, and I also have some problems with my body. Besides, my wife at home also needs me to take care of her. So, I am considering resigning next year.” **(Participant E)**

### Theme 2: coping strategies

**Figure 3:**
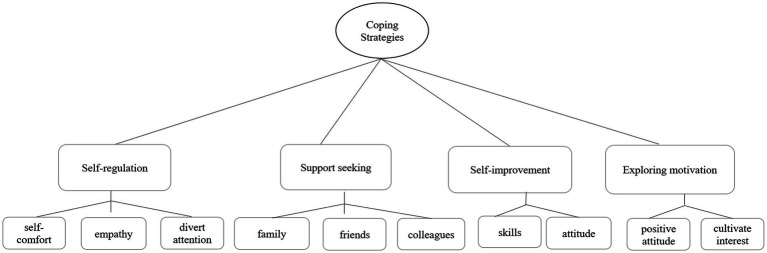
The relationship diagram of the four major themes of coping strategies for older nursing assistants.

Based on the basic work from the first code, Colaizzi’s 7-step analysis method was used to summarize the integrated nodes (Khan, 2014). Four subthemes were identified for older nursing assistants’ coping strategies with turnover intention. The derived relationship diagram is shown in Figure 3.

#### Self-regulation

When reflecting on stress at work and turnover intention, the participants tended to focus on personal coping strategies. Self-regulation, including self-comfort, empathy, and diverting attention, was a common way of coping. For example, some participants empathized with themselves and others, recognizing that everyone would get old and experience health problems. Others found comfort in the fact that there are no easy jobs and that they enjoy what they do. Still, others chose to divert their attention by taking time off to go traveling or shopping.

“Everyone will get old…it is understandable that we lose our tempers because there are so many diseases when we are old” **(Participant B)** (empathy).

“I like what I do. There are no easy jobs” **(Participant F)** (self-comfort).

“When I feel unhappy or angry at work, I will choose to take time off to go traveling or go shopping, and that make me feel much better” **(Participant E)** (divert attention).

#### Support seeking

Family, colleagues and friends were considered an important source of social support and a vital job resource to help balance job demands and positively impacting job satisfaction. Participants often complained to their family members about their work-related stress, and they found their family members to be considerate and kind. Additionally, participants often turned to their friendly colleagues for help and support, and they would help each other out when needed.

“No matter how tired I am, I still complain to my family members because they are always considerate and kind to me.” **(Participant G)**

“I will tell my friendly colleagues and we often help each other.” **(Participant C)**

#### Self-improvement

This subtheme focused on attitudes and skills as coping strategies. Participants recognized that while they may have limited control over job demands, their attitudes and skills could partially protect them from the negative impact of these demands.

“As long as we are technical professional, service enthusiasm, and kind to the older adults, they (older adults and their family members) will trust and be kind to you, too.” **(Participant D)**

Some older nursing assistants were also actively seeking better job opportunities by enhancing their personal competitiveness. They adopted a proactive and positive mindset, which gave them hope for the future. Instead of simply complaining about their current job, they focused on self-improvement to increase their chances of finding better opportunities.

“I want to take a confinement exam to become a *Yuesao* (special care for newborns and mothers), but I cannot just quit my job right away.” **(Participant G)**

#### Exploring motivation

Another motivation for older nursing assistants to continue working was the sense of meaningfulness derived from helping older people and contributing to society. They believed that taking care of others was not only a blessing for the individuals they cared for but also for themselves. This sense of purpose and fulfillment added to their job satisfaction.

Furthermore, some participants mentioned the tangible benefits they gained from their job, such as learning about health preservation and disease prevention. They enjoyed engaging with older adults, engaging in activities like knitting sweaters and learning new skills like embroidery. They also appreciated the knowledge shared by nutritionists, which helped them understand how to match food for different diseases. These tangible benefits added to their job satisfaction and made their work both useful and enjoyable.

“I think that taking care of others is also a blessing for myself (laughs).” **(Participant E)**

“We chat with the older adults and we knit sweaters. One taught me how to embroider shoes!” **(Participant F)**

“A nutritionist in our institution also taught us how to match (food stuff) for different diseases. There is a significant amount of knowledge in this practice, and it is useful and funny to learn.” **(Participant G)**

## Discussion

### Influencing factors of the turnover intention for older nursing assistants

1. Work pay: according to Maslow’s hierarchy of needs, the need for survival is the first level. A study by Xue et al. (2019) demonstrated that earning money to support their family is the main motivation behind why most older nursing assistants pursue their careers. The salary serves as a potential driving force behind their work. Moreover, work pay also provides a good guarantee for a some-good quality of later-life for older nursing assistants when they get retired. According to a cross-sectional study of primary care health workers’ income sources in the Democratic Republic of Congo, older nursing assistants especially have relatively low incomes and long working hours (Maini et al., 2017). With the development of society, the dilemma of the older nursing assistants did not improve, Liao et al. confirmed that this situation is very common in developing countries (Liao et al., 2023). Aspects considered in late-career planning also included personal finances and meaningfulness of work (Brown et al., 2013). Therefore, as the job incomes cannot meet older nursing assistants’ expectations, they were likely to want to change jobs.
2. Environmental factors: older adults are the primary recipients of care in eldercare institutions. However, older nursing assistants employed in eldercare institutions often face complex relationships, with the older adults, family members, nurses, doctors, and other caregivers (Coats et al., 2018). Due to the characteristics of geriatric diseases and the nature of the work, the departmental atmosphere can be relatively depressing and stressful. In addition, some eldercare institution wards are crowded, dirty, and malodorous (O'Connor et al., 2014). Measures should be taken to create healthy and safe work environments for employees, to facilitate successful and sustainable aging-in-workplace.
3. Professional identity: data from the interviews revealed that older nursing assistants lacked the appropriate understanding of the value and importance of their work in caring for older adults. Several participants felt that their profession had low status and was seen as merely serving people. Furthermore, even their relatives often did not understand their profession. Some caregivers hid their careers, believing that they were “shameful” or “faceless.” This finding was consistent with the quantitative studies that were conducted in other regions (Sansoni et al., 2016; Liu et al., 2018). Therefore, to address this issue, efforts should be made to enhance the professional identity of older nursing assistants through education and awareness campaigns. Promoting the importance and value of their work can help improve job satisfaction and retention rates.
4. External motivation: older nursing assistants face competition from other job options that may be seen as more respectable or offer better working conditions. The ability to pursue careers such as *Yuesao* or prolactin division experts, babysitters, or other occupations has provided older adult nurses with multiple alternatives (Lu and Xu, 2023). Moreover, the aforementioned occupations have better working environments, more freedom, and less work pressure compared to older adult nursing care. Further, the aforementioned training methods are more standardized and convenient and the training can be completed in a short period of time and at a lower cost compared to older nursing assistants. These external factors contribute to a higher likelihood of older nursing assistants considering resignation, resulting in an unstable workforce in the field. To address this issue, efforts should be made to improve the working conditions and benefits for older nursing assistants, and provide opportunities for professional development and growth within the field.
5. Work pressure: work pressure is a result of an imbalance between a work environment’s demands and an individual’s ability to cope with these demands. When an individual cannot cope with or minimize all sources of work stress, work stress occurs (Bordignon and Monteiro, 2019). This study established that older nursing assistants are under significant amounts of work pressure. Moreover, the sources of stress include a heavy workload, caregiving problems, and long working hours. Increased work pressure can create suboptimal mental health among caregivers, resulting in conditions such as depression, anxiety, social disorders, and burnout. In consistent with our study findings, in a survey of 308 caregivers in eldercare institutions, Park (2010) found that work pressure in eldercare institutions was caused by interpersonal tension, human resource shortages, job risks, deaths, and other adverse events.

### Suggestions regarding the reduction of the turnover intention for older nursing assistants

This qualitative study provided a theoretical basis for the healthy development of older nursing talent teams. (1) Pension institutions-related: job responsibilities should be standardized among older nursing assistants and competitive salaries and benefits should be provided. Managers should improve the work environment and reduce work-related risks, by providing insurance and other forms of protection, to attract exceptional talent, especially young people, into the field of older adults nursing. These efforts contributed to attracting and retaining outstanding professionals in the field (Nilsson, 2020). (2) Functional departments that strengthen eldercare nurses’ training are imperative. Continuous skill development, which may prevent obsolescence and foster a sense of competence. As suggested in other studies of eldercare (Schalk and Desmette, 2014; Gyllensten et al., 2019), the accumulated experience of older nursing assistants may be valued by the organization through, for example, having older workers mentoring younger colleagues, thereby promoting older employees’ feelings of being needed and appreciated. Moreover, a fair and transparent talent promotion system should be provided. To solve the increasingly serious problem of aging and reduce the brain drain in older adults nursing services, eldercare institutions and government departments such as Labor Bureau and Social Security Bureau should promote older nursing assistants’ participation in training by providing training equipment and training funds (Groenwald and Eldridge, 2020). (3) Older nursing assistants should develop their subjective initiatives and enhance their feelings of self-worth. Personal resources, such as self-efficacy, are important for workers in demanding occupations such as older nursing assistants. Thus, eldercare managers should support older nursing assistants’ efficacy beliefs, such as through realistic encouragement, and highlight employee strengths (Oakman and Howie, 2013). This study found self-efficacy can be influenced by a self-regulating attitude, seeking support, striving for self-improvement, and discovering motivations. Finally, by recognizing and creating opportunities for older nursing assistants to make a positive impact on residents’ quality of life, meaningfulness at work can be promoted, which was found to be a significant motivator to continue working.

### Study limitations

There are several limitations to this study. Due to limitations in resources, our study was conducted in only 8 eldercare institutions in Changsha. Although we used purposive sampling to achieve information saturation, the findings may not be representative of all 32 provinces in China. Additionally, this study did not consider the potential impact of different ethnic groups on the research results. Therefore, when extrapolating the findings, the generalizability of the sample may be somewhat limited. In this initial study, interviews were only conducted with older nursing assistants who were not resigned. It would be beneficial to also interview with other relevant personnel, such as resigned older nursing assistants, eldercare institutional administrators, and service recipients. Interviews with the aforementioned personnel could help to gain a deeper understanding of turnover intentions.

## Conclusion

Older nursing assistants face significant work pressure and lack of social support, which can contribute to their desire to resign from their jobs. Factors such as work pay, environmental factors, professional identity, external motivation, and work pressure influence their intention to resign from their jobs. This qualitative research revealed that lower work pay directly leads to the turnover intention of older nursing assistants. The results also showed that the psychosocial work environment was perceived as stressful and poses long-term health risk, which further impact their decision to leave their jobs. The study also emphasizes that the future health and work ability were key factors in determining nursing assistants’ turnover intention.

To address this issue, effective coping strategies need to be implemented. The current coping strategies for older nursing assistants with the turnover intention include self-regulation, support seeking, self-improvement, and exploring motivation. Our study found that reducing the turnover intention of older nursing assistants also requires united efforts from older nursing institutions, functional departments, as well as older nursing assistants themselves. By providing support and resources, institutions can create an environment that promotes job satisfaction and reduces turnover intention. Functional departments can offer training and mentoring programs to enhance the skills and confidence of older nursing assistants. Finally, nursing assistants themselves can take proactive steps to improve their own well-being and job satisfaction. In conclusion, reducing turnover intention among older nursing assistants requires a collaborative effort from various stakeholders. By addressing factors such as work pay, improving the psychosocial work environment, and implementing effective coping strategies, the turnover intention of older nursing assistants can be minimized, leading to a more stable and satisfied workforce in eldercare institutions.

## Data availability statement

The original contributions presented in the study are included in the article/supplementary material, further inquiries can be directed to the corresponding author.

## Ethics statement

This study has obtained approval from the Ethics Committee of the Second People’s Hospital of Shenzhen (Approval No: 2020081202). The studies were conducted in accordance with the local legislation and institutional requirements. The participants provided their written informed consent to participate in this study.

## Author contributions

YT: Formal analysis, Methodology, Writing – original draft, Writing – review & editing. QZ: Writing – review & editing. HY: Investigation, Writing – review & editing. SS: Resources, Validation, Writing – review & editing. XX: Resources, Visualization, Writing – review & editing. ZY: Funding acquisition, Methodology, Resources, Writing – review & editing.

## Funding

The author(s) declare financial support was received for the research, authorship, and/or publication of this article. This work was supported by the Shenzhen’s Sanming Project Foundation of China (grant no. SZSM201812041).

## Conflict of interest

The authors declare that the research was conducted in the absence of any commercial or financial relationships that could be construed as a potential conflict of interest.

## Publisher’s note

All claims expressed in this article are solely those of the authors and do not necessarily represent those of their affiliated organizations, or those of the publisher, the editors and the reviewers. Any product that may be evaluated in this article, or claim that may be made by its manufacturer, is not guaranteed or endorsed by the publisher.
